# The force of the myosin motor sets cooperativity in thin filament activation of skeletal muscles

**DOI:** 10.1038/s42003-022-04184-0

**Published:** 2022-11-18

**Authors:** Marco Caremani, Matteo Marcello, Ilaria Morotti, Irene Pertici, Caterina Squarci, Massimo Reconditi, Pasquale Bianco, Gabriella Piazzesi, Vincenzo Lombardi, Marco Linari

**Affiliations:** 1 PhysioLab, University of Florence, Florence, Italy; 2 Department of Biology, University of Florence, Florence, Italy; 3 Department of Experimental and Clinical Medicine, University of Florence, Florence, Italy

**Keywords:** Physiology, Biophysics

## Abstract

Contraction of striated muscle is regulated by a dual mechanism involving both thin, actin-containing filament and thick, myosin-containing filament. Thin filament is activated by Ca^2+^ binding to troponin, leading to tropomyosin displacement that exposes actin sites for interaction with myosin motors, extending from the neighbouring stress-activated thick filaments. Motor attachment to actin contributes to spreading activation along the thin filament, through a cooperative mechanism, still unclear, that determines the slope of the sigmoidal relation between isometric force and pCa (−log[Ca^2+^]), estimated by Hill coefficient *n*_H_. We use sarcomere-level mechanics in demembranated fibres of rabbit skeletal muscle activated by Ca^2+^ at different temperatures (12–35 °C) to show that *n*_H_ depends on the motor force at constant number of attached motors. The definition of the role of motor force provides fundamental constraints for modelling the dynamics of thin filament activation and defining the action of small molecules as possible therapeutic tools.

## Abstract (web summary)

Sarcomeres of Ca^2 +^ -activated demembranated fibres of rabbit soleus muscles show that the stress exerted by each motor protein on the thin filament determines the cooperativity in thin filament activation.

## Introduction

**Fig. 1:**
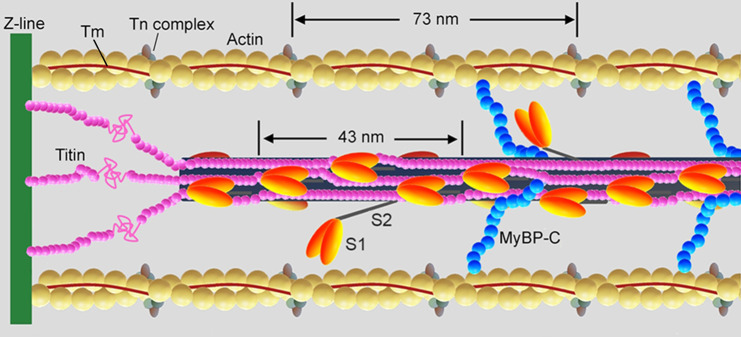
Schematic representation of the half-sarcomere protein assembly. Shown are actin (yellow), tropomyosin (Tm, brown) and troponin complex (Tn, light and dark grey and violet) on the thin filament. On the thick filament (black) most of the S1 globular head domains of myosin (orange) lie tilted back (OFF state) and a few heads (two in the scheme) move away with tilting of their S2 tail domain (ON state); the MyBP-C (blue) lies on the thick filament with the C-terminus and extends to thin filament with the N-terminus. Titin (pink) in the I-band connects the Z line at the end of the sarcomere (green) to the tip of the thick filament and in the A-band runs on the surface of the thick filament up to the M-line at the centre of the sarcomere. Adapted from Fig. 1 in ref. ^78^ (permission from Springer Nature).

Contraction in striated muscle is driven by myosin motors, extending from the thick filaments and pulling on the neighbouring thin, actin-containing, filaments, through cyclical ATP driven interactions. In the resting muscle, contraction is prevented by tropomyosin (Tm), a regulatory protein in the thin filament that sterically blocks myosin binding sites on actin (Fig. 1).

Nerve action potentials, transmitted via the neuromuscular junction to the muscle cell (fibre), trigger Ca^2+^ release from sarcoplasmic reticulum and rapid activation of the thin filament by Ca^2+^ binding to troponin C (TnC, the Ca^2+^ sensor of the Tn complex, the other regulatory protein in the thin filament) followed by azimuthal displacement of Tm that exposes the actin sites for the interaction with the myosin motors^1^. On the other hand, in the resting muscle most of myosin motors lie on the surface of the thick filament kept folded back towards the centre of the sarcomere (OFF state, Fig. 1) by head-head and head-tail interactions, unable to bind to actin and hydrolyse ATP^2–5^. In loaded contractions the stress generated by a few constitutively ON motors on the thick filament moves the main population of OFF motors away from the thick filament (ON state) making them available for actin binding^6–9^. This process is likely modulated by accessory proteins on the thick filament like the Myosin Binding Protein-C (MyBP-C) or cytoskeleton proteins like titin and their degree of phosphorylation (Fig. 1)^10^, but in relaxed demembranated fibres the ordered OFF conformation of the myosin motors is known to be perturbed also by lowering temperature below the physiological value^11–14^.

Thin filament activation by Ca^2+^ binding to TnC progresses through the activation of Regulatory Units (RU) composed, for each strand of the double stranded helix, of 7 actin monomers (5.5 nm each in diameter), 1 Tn complex and 1 Tm (Fig. 1). However, Ca^2+^-activation can spread beyond a RU for head-to-tail interactions between consecutive Tm’s^15^, which suggests a way for the cooperative thin filament activation. Biochemical and structural evidence indicates that full actin site availability implies strong myosin attachment to actin^16–20^ supporting the two-step steric-blocking model of thin filament activation:^21^ the first step, triggered by Ca^2+^ binding to TnC, is responsible for a partial displacement of Tm that within a RU leads from the blocked state, in which myosin can only weakly interact with actin, to the closed state that allows weak-to-strong binding transition of myosin; the second step, triggered by strong binding of myosin, promotes further Tm displacement leading to an open state that may in turn provide the cooperative mechanism that spreads thin filament activation^22,23^. Cooperativity determines the slope of the sigmoidal relation between force and pCa in the demembranated fibre, estimated by Hill coefficient *n*_H_ (see Methods).

According to a classical view of the role of motors in cooperative activation, for a given number of Ca^2+^-Tn RU’s, related to [Ca^2+^] through the affinity of TnC for Ca^2+^, the value of *n*_H_ underlies the number of strongly attached motors (from here on simply called attached motors) and the other way around^22–25^. Accordingly, modulation of the Ca^2+^ affinity by TnC replacement with mutant TnC shifts the force-pCa relation without significant effects on the slope of the relation^26,27^. In this respect, however, a contradiction emerges with the so far unexplained finding that *n*_H_ of the force-pCa relations of cardiac and slow skeletal myocytes in the presence of omecamtiv mecarbil (OM) is quite low notwithstanding the high fraction of attached motors^28–30^. OM is an activator of the β/slow isoform of myosin, which increases Ca^2+^ sensitivity by increasing the affinity of myosin for actin but also inhibits the execution of the working stroke and thus force generation and shortening, while the motor remains strongly bound to actin.

Here we use fast sarcomere-level mechanics in Ca^2+^-activated demembranated fibres of rabbit soleus muscle to determine the average force of the motor (*F*_0_) under different interventions and define if the stress exerted by the motor on the thin filament has a role in cooperative thin filament activation beyond the steric effect of motor attachment. To this end we study how changing temperature, an intervention that in fast muscle fibres is known to change the force of the myosin motor without changing the number of attached motors^31–34^, affects the characteristics of the force-pCa relation. We find that in Ca^2+^-activated fibres of rabbit soleus the rise in temperature from 12 to 35 °C, which does not change the number of attached motors and increases *F*_0_ by 50%, increases *n*_H_ in proportion. The same increase in temperature in the presence of 1 μM OM, which per se reduces *F*_0_ to ½ by preventing the execution of the force-generating step in the OM-bound motors (from here on OM-motors, 50% of the total attached motors^30^), doubles *F*_0_, without significant increase in *n*_H_. These results suggest that the motor force plays a role in the cooperativity of thin filament activation. Consequently, the classical steric-blocking model must be integrated with the concept that the displacement of Tm and the availability of neighbouring actin sites are under the dynamic control of the motor force. This mechanism is specifically blocked by attachment of motors with force inhibited by OM. Extending the *n*_H_–*F*_0_ relation in control to higher *F*_0_ values with data from fast skeletal muscle reveals that the cooperative mechanism that relates *n*_H_ to *F*_0_ is unique for the two muscle types, as expected if it specifically depends on the myosin motor force, and saturates at high values of *F*_0_ according to a two-step process with Michaelis-Menten kinetics.

## Results

### The effect of temperature on the force-pCa relation and on the force of the myosin motor

**Fig. 2:**
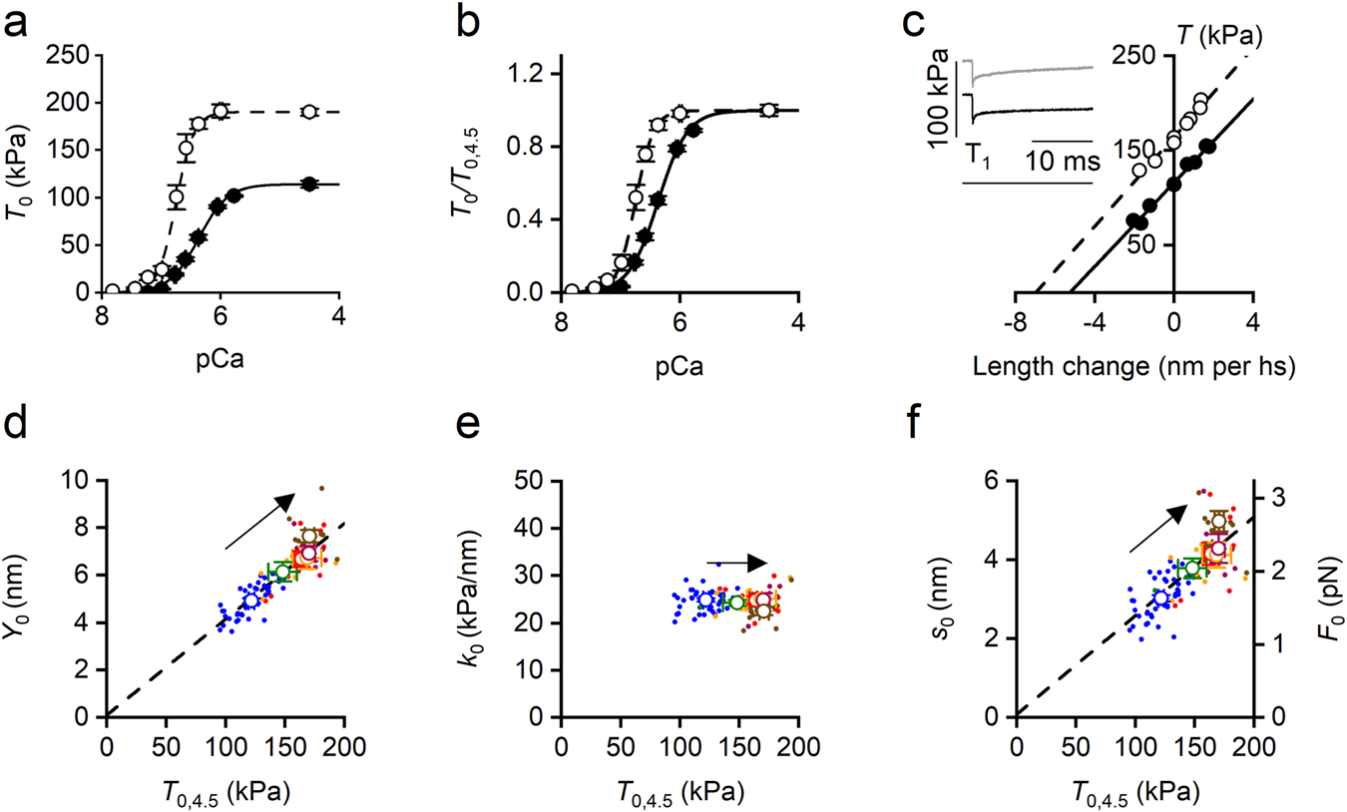
Effect of temperature on the relevant mechanical parameters of Ca^2+^-activated skinned soleus fibre. **a** Force-pCa relations interpolated with the Hill equation (see SI Methods). Filled circles and continuous line, 12 °C; open circles and dashed line, 35 °C. *T*_0_ in absolute units (kPa). **b** As in a with *T*_0_ relative to *T*_0_ at pCa 4.5 (*T*_0,4.5_). **c** *T*_1_ relations at 12 °C (filled symbols) and 35 °C (open symbols) at pCa 4.5. Lines are first order regression equations fitted to the data at 12 °C (continuous) and 35 °C (dashed). In the inset sample records of the force response to the small step release are superimposed to show the force attained at the end of the step (*T*_1_). From top to bottom force at 35 °C, at 12 °C and force baseline. **d** Dependence on temperature -modulated *T*_0,4.5_ of *Y*_0_ (half-sarcomere strain) and fit of pooled data with a first order regression equation (dashed line, slope 0.040 ± 0.003 nm/kPa, ordinate intercept 0.10 ± 0.38 nm). **e** Dependence on *T*_0,4.5_ of *k*_0_ (half-sarcomere stiffness). **f** Left ordinate: dependence on *T*_0,4.5_ of *s*_0_ (motor strain) and linear fit of pooled data (dashed line, slope 0.025 ± 0.003 nm/kPa and ordinate intercept 0.08 ± 0.37 nm). Right ordinate: dependence on *T*_0,4.5_ of *F*_0_ (motor force). The corresponding parameters of the linear fit are 0.014 ± 0.001 pN/kPa (slope) and 0.04 ± 0.20 pN (ordinate intercept). In **d** and **f** the slopes are significantly different from zero (*P* always <0.005) and the ordinate intercepts are not significantly different from zero (*P* always >0.75). The arrows in panels **d**–**f** indicate the direction of the rise in temperature from 12 to 35 °C; the colours refer to different temperatures: 12 °C, blue; 17 °C, green; 20 °C, red; 25 °C, orange; 30 °C, purple; 35 °C, brown. In **a** and **b**, data are mean values (± SEM) from 5 fibres. In **c** data from one fibre: fibre length, 3.23 mm; length of the segment under the striation follower, 0.72 mm; average sarcomere length, 2.30 μm; CSA, 4210 μm^2^. In **d**–**f**, filled circles are pooled data and open circles the mean values (± SEM) from 31 fibres.

Increase in temperature from 12 to 35 °C increases the isometric force (*T*_0_) of the soleus fibre at any [Ca^2+^], as shown by the force-pCa relations in Fig. 2a (symbols and lines, data and interpolated Hill equation respectively; filled circles and continuous line, 12 °C; open circles and dashed line: 35 °C). The relation at 35 °C is steeper, as it better emerges when *T*_0_ is plotted as a fraction of *T*_0,4.5_ (the maximum value measured at pCa 4.5) at each temperature (Fig. 2b). The underlying cooperativity index (estimated by the parameter *n*_H_ of Hill equation, see Methods) increases with temperature and so does the Ca^2+^-sensitivity of thin filament, as shown by the leftward shift of the pCa at which *T*_0_ attains 0.5*T*_0,4.5_ (pCa_50_, Fig. 2b). In the temperature range 12–35 °C, *T*_0,4.5_ increases from 127 ± 5 to 183 ± 3 kPa, *n*_H_ increases from 1.97 ± 0.07 to 3.09 ± 0.20 and pCa_50_ increases from 6.38 ± 0.05 to 6.67 ± 0.09 (that is [Ca^2+^]_50_ reduces from 0.42 μM to 0.21 μM) (Table 1).

**Table 1:** Effect of temperature on the relevant mechanical parameters listed on the first row.

	Temperature (°C)	*T*_0,4.5_ (kPa)	*s*_0_ (nm)	*F*_0_ (pN)	*n*_H_	*pCa*_50_
soleus fibre						
control						
	12.3 ± 0.1 (14)	127 ± 5	3.16 ± 0.12	1.71 ± 0.06	1.97 ± 0.07	6.38 ± 0.05
	17.3 ± 0.1 (4)	148 ± 12	3.78 ± 0.25	2.04 ± 0.14	2.42 ± 0.09	6.54 ± 0.06
	25.2 ± 0.1 (4)	165 ± 12	4.53 ± 0.20	2.45 ± 0.11	3.15 ± 0.13	6.55 ± 0.03
	35.5 ± 0.1 (5)	183 ± 3	5.34 ± 0.32	2.59 ± 0.19	3.09 ± 0.20	6.67 ± 0.09
1 µM OM						
	12.2 ± 0.1 (5)	69 ± 3	1.53 ± 0.05	0.83 ± 0.06	0.79 ± 0.09	7.17 ± 0.09
	25.2 ± 0.1 (5)	117 ± 14	2.75± 0.34	1.48 ± 0.18	0.96 ± 0.13	7.21 ± 0.10
	35.6 ± 0.1 (5)	134 ± 14	3.11 ± 0.30	1.68 ± 0.16	1.17 ± 0.08	7.16 ± 0.08
psoas fibre						
	6.0 ± 0.1 (4)	135 ± 6	1.71 ± 0.11	2.90 ± 0.18	3.51 ± 0.22	5.89 ± 0.05
	12.1 ± 0.1 (12)	219 ± 11^#^	2.86 ± 0.16^#^	4.87 ± 0.27	3.92 ± 0.21	6.00 ± 0.13
	25.0 ± 0.1 (6)	317 ± 14^#^	4.13 ± 0. 19^#^	7.02 ± 0.32	4.81 ± 0.22	6.46 ± 0.06

Data for soleus fibres in control and in the presence of 1 µM OM. For comparison also data for psoas fibres are reported (from ref. ^33^ and new data). Data are mean ± SEM. In brackets the number of fibres used for each temperature. ^#^ mean calculated by pooling data from new experiments with data from ref. ^33^. In each of the three cases the statistical significance of the temperature effect is tested with one-way ANOVA as detailed in Supplementary Table 2. The soleus values in control of *F*_0_ and *n*_H_ at 35.6 °C are not significantly different from the corresponding psoas values at 6.0 °C (*P* = 0.24 and 0.16 respectively).

According to the simplified mechanical model of the half-sarcomere (see Methods and Supplementary Fig. 1), the half-sarcomere (hs) stiffness of the Ca^2+^-activated fibres, *k*_0_, can be used to calculate the strain and force of the motor and the compliance of the myofilaments^33,35^. To this end, step perturbations in length imposed on the fibre at *T*_0_ (Supplementary Fig. 2a–c, temperature 12 °C) were used to build the *T*_1_ relations at different pCa ranging from 6.8 (Supplementary Fig. 2d, triangles) to 4.5 (circles). The slope and the abscissa intercept of the *T*_1_ relations estimate *k*_0_ and the hs strain (*Y*_0_) respectively. *k*_0_ and *Y*_0_ increase with the Ca^2+^-dependent increase of *T*_0_, though less than in proportion with *T*_0_ (Supplementary Fig. 3a, b, respectively), as a consequence of the underlying contributions of two mechanical elements in series (Supplementary Fig. 1): (i) the array of myosin motors, the stiffness of which (*e*_0_) increases in proportion to the Ca^2+^-dependent increase of *T*_0_ (Supplementary Fig. 3c), underpinning a proportional increase in the fraction of attached motors, and (ii) the myofilaments, the equivalent compliance of which (*C*_f_) is estimated as the slope of the *Y*_0_-*T*_0_ relation (Supplementary Fig. 3b and Eq. 3 in Methods)^33,35^.

In the soleus fibre, stiffness measurements can be reliably done at temperatures as high as 35 °C, because the quick force recovery following a step is much slower than in the fast, psoas fibre and the truncation of *T*_1_ response by quick recovery is anyway minimum (Fig. 2c). The relation between hs stiffness and Ca^2+^-dependent force and the ensuing analysis of the half-sarcomere compliance are repeated at 35 °C and are superimposed on those at 12 °C in Supplementary Fig. 4a–c (filled circles 12 °C, open circles 35 °C). The analysis makes evident that the motor strain *s*_0_ (the ordinate intercept of the *Y*_0_–*T*_0_ relation, Supplementary Fig. 4b) is the parameter that accounts for both the 40% increase in force between 12 and 35 °C and the apparent reduction, at a given force, of the stiffness of the half-sarcomere (Supplementary Fig. 4a) and the motor array (Supplementary Fig. 4c).

The mechanism underlying the potentiating effect of temperature on *T*_0,4.5_ is analysed at six temperatures in the range 12–35 °C in Fig. 2d–f. It can be seen that the increase in *T*_0,4.5_ is accounted for by a proportional increase in hs strain (*Y*_0_, Fig. 2d) without significant change in hs stiffness (*k*_0_, 24.4 ± 0.4 kPa/nm estimated in the whole temperature range, Fig. 2e). Consequently, also *e*_0_, obtained according to Eq. 2 by subtracting from the hs compliance (1/*k*_0_) the contribution of filament compliance (*C*_f_, 13.66 ± 3.02 nm/MPa, estimated in the whole temperature range as in Supplementary Fig. 3b) is constant independent of temperature (39.9 ± 0.9 kPa/nm), indicating that the fraction of the attached motors does not change with temperature. *s*_0_, instead, increases in proportion to *T*_0,4.5_ (Fig. 2f, left ordinate) as shown by the first order equation fit to data (line) that exhibits an ordinate intercept not significantly different from zero.

It has been previously shown that under the same experimental conditions used here (temperature 12 °C, sarcomere length 2.3–2.5 μm, ionic strength 190 mM) the fraction of attached motors at *T*_0,4.5_ in soleus fibres is 0.47^35^. With this value and the known density of myosin motors per half thick filament (1.58*10^17 ^m^-2^^35^,) the stiffness of the attached myosin motor (ε_0_) can be calculated from *e*_0_ and is [39.87/(0.47*1.58*10^17 ^m^-2^) =] 0.54 ± 0.01 pN/nm. The average force per myosin motor *F*_0_ can be calculated at each temperature by the product *s*_0_*ε_0_. *F*_0_ increases with *s*_0_ from 1.7 pN at 12 °C to 2.6 pN at 35 °C (Fig. 2f, right ordinate and Table 1).

### The effect of temperature in the presence of 1 μM OM

**Fig. 3:**
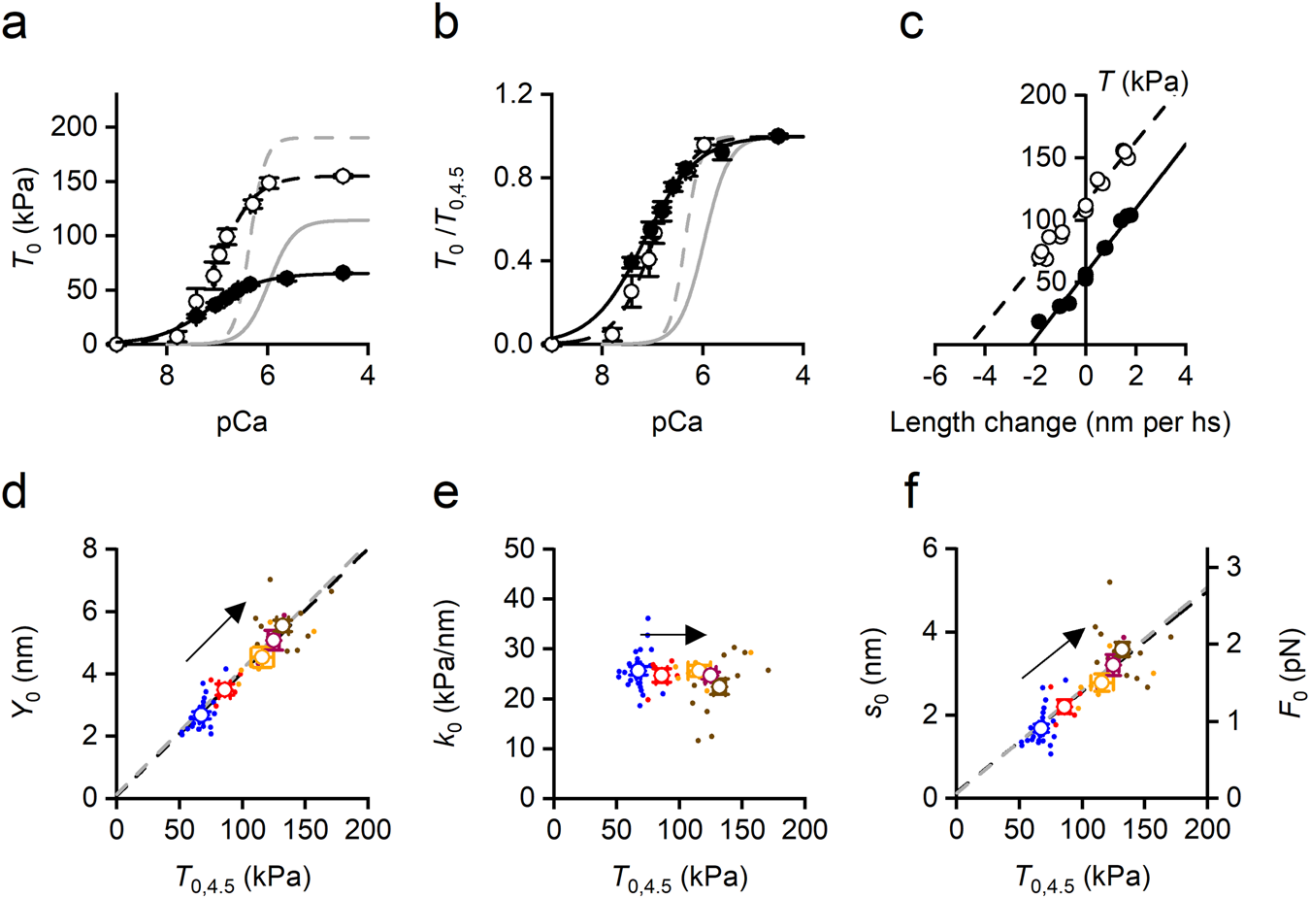
Effect of temperature on the relevant mechanical parameters of Ca^2+^-activated skinned soleus fibre in the presence of 1 μM OM. OM relations (black symbols and lines) are compared with relations in control from Fig. 2 (grey lines). **a** Force-pCa relations interpolated with the Hill equation at 12 °C (filled circles and continuous line) and 35 °C (open circles and dashed line). *T*_0_ in absolute units (kPa). **b** As in a with *T*_0_ relative to *T*_0_ at pCa 4.5 (*T*_0,4.5_). **c** *T*_1_ relations at 12 °C (filled symbols) and 35 °C (open symbols). Lines are first order regression equations fitted to the data at 12 °C (continuous) and 35 °C (dashed). **d** Dependence on temperature modulated *T*_0,4.5_ of *Y*_0_ (half-sarcomere strain,) and fit of pooled data with a first order regression equation (dashed line, slope 0.040 ± 0.003 nm/kPa, ordinate intercept 0.09 ± 0.27 nm). **e** Dependence on *T*_0,4.5_ of *k*_0_ (half-sarcomere stiffness). **f** Left ordinate: dependence on *T*_0,4.5_ of *s*_0_ (motors strain) and linear fit of pooled data (dashed line, slope 0.024 ± 0.003 nm/kPa and ordinate intercept 0.16 ± 0.26 nm). Right ordinate: dependence on *T*_0,4.5_ of *F*_0_ (motor force). The corresponding parameters of the linear fit are 0.013 ± 0.001 pN/kPa, slope and 0.08 ± 0.14 pN, ordinate intercept. In **d** and **f** the slopes are significantly different from zero (*P* always <0.005) and the ordinate intercepts are not significantly different from zero (*P* always >0.5). The arrows indicate the direction of the rise in temperature from 12 to 35 °C; the colours refer to different temperatures: 12 °C, blue; 20 °C, red; 25 °C, orange; 30 °C, purple; 35 °C, brown. In **a** and **b**, data are mean values (±SEM) from 7 fibres. In **c** data from one fibre: fibre length, 4.75 mm; length of the segment under the striation follower, 0.63 mm; average sarcomere length, 2.31 μm; CSA, 2800 μm^2^. In **e** and **f** filled circles are pooled data and open circles the mean values (±SEM) from 17 fibres.

In the presence of 1 μM OM the rise in temperature from 12 to 35 °C increases the isometric force of the soleus fibre at any [Ca^2+^] and the slope of the force-pCa relation (Fig. 3a and b: 12 °C, filled circles and interpolated black continuous line; 35 °C, open circles and interpolated black dashed line; in b, data are plotted relative to *T*_0,4.5_ at each temperature). At either temperature, in OM with respect to control (grey lines from black lines in Fig. 2a and b respectively), *T*_0,4.5_ is depressed, *n*_H_ is smaller and pCa_50_ is larger (see also Table 1). Moreover, *n*_H_ increases with temperature (12–35 °C, *P* < 0.05) in OM as in control, while pCa_50_ does not change significantly (*P* > 0.9, Table 1).

Stiffness measurements in the presence of 1 μM OM (Fig. 3c) show that, as in control, the slope of the *T*_1_ relation at *T*_0,4.5_ is the same at 12 °C (filled circles and continuous line) as at 35 °C (open circles and dashed line). The half-sarcomeres strain, *Y*_0_, determined at *T*_0,4.5_ at five temperatures in the range 12–35 °C, increases in proportion with *T*_0,4.5_ (Fig. 3d: circles, data points and black line, first order equation fit), like in control (grey line from Fig. 2d), so that the half-sarcomere compliance (the slope of the relation, 39.7 ± 2.6 nm/MPa) is not significantly different from that in control (40.5 ± 2.6 nm/MPa, Fig. 2d, *P* > 0.8). Consequently, the hs stiffness, *k*_0_, is constant in the whole temperature range (Fig. 3e, average 24.6 ± 0.6 kPa/nm) and not significantly different from that in control (Fig. 2e, *P* > 0.7).

*s*_0_ and *C*_f_ at any given temperature have been estimated in the presence of 1 μM OM by applying the same analysis as in control (see Supplementary Fig. 3d for temperature 12 °C and Supplementary Fig. 4d for the effect of rising temperature to 35 °C) to hs stiffness measurements in fibres activated at different [Ca^2+^]: *s*_0_ increases from 1.66 ± 0.14 nm to 3.03 ± 0.28 nm with temperature while *C*_f_ is constant.

The effect of temperature on *T*_0,4.5_ in the range 12–35  °C is explained by proportional changes in *s*_0_ (Fig. 3f, left ordinate), while the stiffness of the motor array *e*_0_, calculated from *k*_0_ (Fig. 3e) by subtracting the contribution of filament compliance, is independent of temperature (39.1 ± 0.6 kPa/nm) and practically identical to the value in control (39.9 ± 0.9 kPa/nm), indicating that in either condition the fraction of attached motors underpinning *T*_0,4.5_ is the same as reported in ref. ^30^. The fraction of attached motors that are bound to OM (50% in 1 μM OM at 12 °C according to ref. ^30^) does not generate force^29,30^. Thus, in 1 μM OM at 12 °C, while the total fraction of attached motors, estimated by *e*_0_, is the same as in control, *T*_0,4.5_ (69 ± 3 kPa) is ½ of that in control (127 ± 5 kPa, Table 1) under the condition that ε is the same^30^. Temperature dependent changes of *T*_0,4.5_ in either case are totally accounted for by proportional increases in average *s*_0_ (Fig. 3f left ordinate, compare grey (control) and black (OM) dashed lines) and thus in *F*_0_ (= ε*s_0_, right ordinate).

### The relation between *n*_H_ and motor force

Both changing temperature and addition of OM are effective interventions for modulating the isometric force of Ca^2+^-activated fibres of rabbit soleus through mechanisms that change the average force of the myosin motor without changing the number of attached motors. Under this condition it is possible to test the specific role of the force of the motor in the cooperative activation of the thin filament without the confounding effect that changes in number of attached motors could introduce through the steric-blocking mechanism that links motor attachment and Tm displacement.

**Fig. 4:**
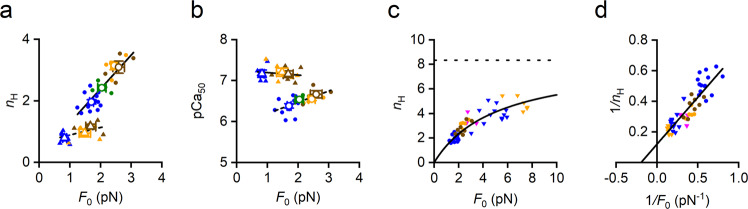
Dependence of *n*_H_ and pCa_50_ on *F*_0_. **a** *n*_H_−*F*_0_ relations for the soleus fibres in control (circles) and in the presence of 1 μM OM (triangles). Filled symbols, pooled data from twenty-three fibres; open symbols, mean values (±SEM) at each temperature. Different colours refer to different temperatures: 12 °C, blue; 17 °C, green; 25 °C, orange; 35 °C, brown. Lines are first order regression equations fitted to pooled data (continuous, control; dashed, OM). The slope and the ordinate intercept of the regressions are: control, 1.10 ± 0.15 pN^-1^ and 0.19 ± 0.32 respectively; 1 μM OM, 0.25 ± 0.13 pN^-1^ and 0.65 ± 0.18 respectively. **b** pCa_50_−*F*_0_ relations in the soleus fibre in control (circles) and in the presence of 1 μM OM (triangles) from the same force-pCa relations as in a. Lines are the first order regression equations fitted to pooled data (continuous, control; dashed, OM). The slope and the ordinate intercept of the regressions are: control, 0.28 ± 0.07 pN^-1^ and 5.92 ± 0.14 respectively; 1 μM OM, −0.06 ± 0.11 pN^-1^ and 7.26 ± 0.15 respectively. **c** Superimposed *n*_H_–*F*_0_ relations in control from the soleus fibres (circles from **a**) and from psoas fibres (reverse triangles, pooled from ref. ^33^ and new data for a total of twelve fibres). Different colours of reverse triangles refer to different temperatures: 6 °C, magenta; 12 °C, blue; 25 °C, orange. The continuous line is calculated from the linear fit of the relation of reciprocal of pooled *n*_H_−*F*_0_ data (continuous line in **d**), the horizontal dashed line is drawn starting from the ordinate that is the reciprocal of the ordinate intercept in **d**. **d** Same data as in **c** plotted as reciprocals. The line is the first order regression equation fitted to the pooled data (slope, 0.61 ± 0.05 pN; ordinate intercept, 0.12 ± 0.02; abscissa intercept -0.20 ± 0.04 pN^-1^).

Temperature-dependent changes of *n*_H_ in the control force-pCa relations are linearly related to *F*_0_ (Fig. 4a: filled circles pooled data, open circles average at each temperature; blue 12 °C, green 17 °C, orange 25 °C, brown 35 °C). The first order equation fit to pooled data (continuous line) gives a slope of 1.10 ± 0.15 *n*_H_ units pN^-1^. In OM the *n*_H_-*F*_0_ relation in the same range of temperatures (triangles, same colour code as control) exhibits a much smaller sensitivity on *F*_0_ (the linear fit to pooled data, dashed line, gives a slope of 0.25 ± 0.13 *n*_H_ units pN^-1^) and is shifted downward by at least 1 *n*_H_ unit.

The pCa_50_ in the same temperature range appears to be related to *F*_0_ in control (Fig. 4b, circles, same symbols and colour code as in a) but not in OM (triangles), in which case the data are shifted upward by at least 0.5 pCa units (see also Table 1). The much larger Ca^2+^ sensitivity characterising the force-pCa relation in OM is in agreement with structural evidence from both fluorescent probes^36^ and X-ray diffraction^37^ that OM binding to myosin promotes the release of a fraction of motors from the resting OFF state even at low [Ca^2+^]. The increase of pCa_50_ in OM is accompanied by a marked reduction in cooperativity, shown by the downward shift of *n*_H_ data and blunting of the *n*_H_ sensitivity to *F*_0_ (triangles in Fig. 4a). These features make evident that the inhibitory effect of OM on *n*_H_ is beyond that expected from the reduction of the average value of *F*_0_ and must be explained by the specific mechanism by which the average *F*_0_ is reduced in OM with respect to control, namely the loss of force generating capability by OM-motors^29,30^.

### A mechanistic model that explains the dependence of cooperativity on *F*_0_ and the inhibition of cooperativity by OM

The qualitative model presented here serves as a proof-of-concept for the hypothesised mechanism by which *F*_0_ determines the degree of cooperativity of thin filament activation. To keep the model as simple as possible (i) the 3D lattice of thin and thick filaments at full filament overlap is reduced to one thick filament facing a single stranded thin filament and the number of myosin dimers per RU is assumed to be two according to the calculation detailed in Supplementary Note 1, (ii) 12 RU’s are considered as a minimum representative number, and (iii) the description of the events is limited to a [Ca^2+^] (10^-7^ M) just above the threshold of the force-pCa relation (the minimum [Ca^2+^] for a detectable rise of active force), at which only two RU’s are assumed to be activated by Ca^2+^.

The basic assumptions concerning the cooperative mechanism and its inhibition by OM are (i) the longitudinal extent of Tm displacement and thus the number of neighbouring actin monomers that become available (*n*_A_) on either side of the monomer strongly bound to a motor depend on motor force *F*, which corresponds to the average motor force *F*_0_ determined experimentally in control (Table 1) and, at a given temperature, is the same for all force generating motors, independent of the presence/absence of OM in the solution. *n*_A_ depends on *F* according to the expression *n*_A_ = 4 + 2.6*F_._ At 12 °C (*F* = 1.7 pN) *n*_A_ ∼ 8 and at 35 °C (*F* = 2.6 pN) *n*_A_ ∼ 11. OM-motors have *F* = 0 and thus, following the attachment of an OM-motor, *n*_A_ = 4. (ii) a motor in the ON state attaches to the nearby actin monomer if it is made available by Tm displacement caused by either Ca^2+^-activation of the RU or the cooperative action of a nearby attached motor; (iii) the attachment of an OM-motor in a region where also other attached motors give their contribution to thin filament activation inhibits the action of the force generating motors, limiting Tm displacement and *n*_A_ according to the action of the OM-motor.

**Fig. 5:**
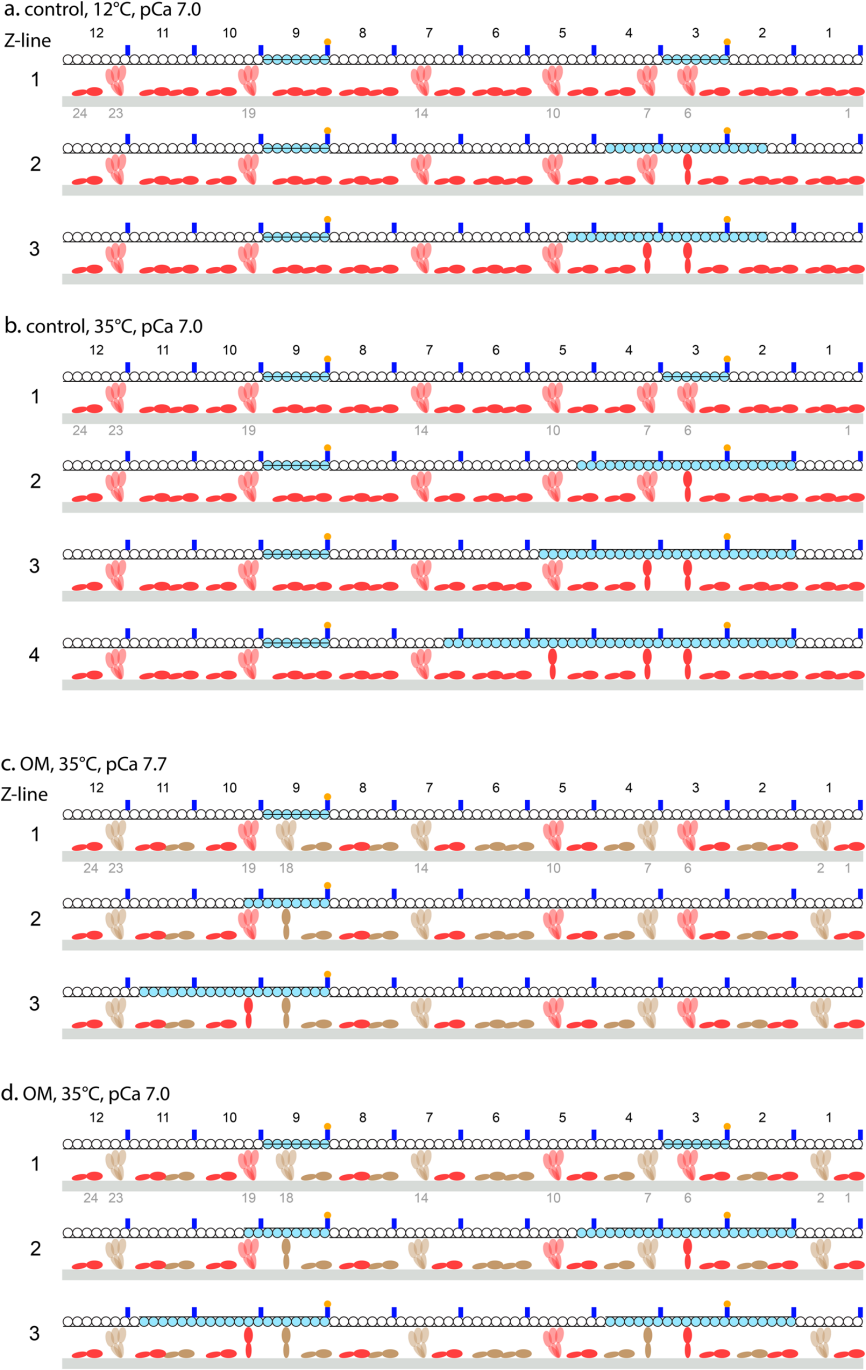
Mechanistic model to explain the dependence of the cooperativity (*n*_H_) on the force per myosin motor (*F*_0_) and the inhibitory effect of OM. In each line of **a**–**d**: the upper filament is the thin filament with monomers (circles) either blocked (white) or available for myosin attachment (cyan), Tm, black line shifted according to the three states (down blocked, middle closed, up open). Tn complex, blue, Ca^2+^, yellow. The filament is reduced to 12 RU’s for simplicity (identified by black numbers from 1 to 12 in the Z-ward direction); the lower filament is the thick filament (grey) carrying two myosin molecules per RU (red without OM, brown OM-bound) identified by green numbers from 1 to 24 in the Z-ward direction. The 18 molecules lying on the surface of the thick filament represent those not available for actin attachment, the 6 that emerge from the backbone are those that attach to an actin site once it is made available by Tm displacement (black line moves up) caused by either Ca^2+^- activation of the RU or the cooperative action of a nearby attached motor. In each panel, line 1 represents the starting conditions at a given pCa and the subsequent lines represent the sequence of events triggered by activation and myosin attachment until a steady state for that pCa is attained. **a** and **b**, in control solution at pCa 7 and 12 °C and 35 °C respectively. **c** and **d**, in the presence of 1 μM OM at 35 °C and pCa 7.7 and 7 respectively.

Under the conditions of the present experiments, we assume that thin filament activation and its cooperativity are not influenced by mechanosensing-based myosin filament activation^6^. In fact, the temperature jump technique exploited to elicit contractions preserving the sarcomere order and the possibility to apply fast mechanical methods to a selected population of sarcomeres requires that the activating solution is first equilibrated into the fibre at 1 °C^33^, at which the myosin filament structure of the relaxed skinned fibres has already undergone a substantial transition to the ON/disordered state^14^. The absence of a significant effect of myosin filament activation in the present experiments appears evident from the lack of asymmetry in the force-pCa relation (Fig. 2b and Supplementary Fig. 5), which underpins only one mechanism for cooperativity in actin filament activation^38^, as explained in detail in Discussion. Accordingly, the fraction of motors that in control is able to attach to the activated actin monomers at [Ca^2+^] just above the threshold in Fig. 5a, b is assumed constant and = (6/24) ¼, only a bit less than the fraction attached in the steady isometric contraction as suggested by the classical duty ratio value (∼1/3). The further final progression in myosin filament activation due to OM (Fig. 5c, d) is assumed to explain the leftward shift of the threshold of the force-pCa relation. In this respect the finding that in control solution the threshold does not change with the test temperature (Fig. 2b, but see also ref. ^24^) confirms that the degree of myosin filament activation is pre-determined by the low temperature before the temperature-jump activating protocol.

The dependence of cooperativity on *F*_0_ in control is explained by the model by comparing the sequence of events at 12 °C (Fig. 5a) and 35 °C (Fig. 5b) at pCa 7, at which only two RU’s of the twelve considered are Ca^2+^-activated (line 1). The step-by-step process at either temperature is described in detail in Supplementary Note 1. The final outputs are that two motors are attached and develop force at 12 °C (line 3 in a), while at 35 °C three motors are attached and develop force (line 4 in b).

The explanation (at 35 °C) of the double action of OM, that is the leftward shift of the threshold of the force-pCa relation with increase in pCa_50_ and the drop in *n*_H_, emerges from the sequence of events described in Fig. 5c and d. The increased spatial frequency of all motors in the ON state for the contribution of OM-motors anticipates the attachment/force generation by the first motor to pCa 7.7 (Fig. 5c), at which only one RU is activated (line 1). In this way OM induces a leftward shift of the threshold of the force pCa-relation that accounts for the observed increase in pCa_50_ (Fig. 3b). At pCa 7 (Fig. 5d), a sub-saturating pCa at which the number of activated RU’s is 2 (line 1) and the observed force-pCa points in OM approach those in control (Fig. 3b), *n*_H_ is reduced by the interposition of zero force OM-motors which specifically limit the propagation of thin filament activation by force-generating motors, as described in detail in Supplementary Note 1. The final output is that in the presence of OM, at pCa 7 and 35 °C, the steady state force is that of two motors (line 3 in d), less than in control at the same pCa and temperature (3 motors, line 4 in b), in spite of the leftward shift of the threshold.

## Discussion

### The relation between *n*_H_ and *F*_0_

We find that, in Ca^2+^-activated demembranated fibres of slow skeletal muscle (rabbit soleus), under conditions in which the number of strongly bound myosin motors is kept constant, the cooperativity in thin filament activation expressed by Hill coefficient *n*_H_ is linearly related to the force of the myosin motor *F*_0_. Both *n*_H_ and *F*_0_ are relatively small compared to the corresponding values in fast skeletal muscle of the same animal (rabbit psoas) expressing the fast Heavy Mero Myosin (HMM) isoform, which develops a force *F*_0_ up to 7 pN at near physiological temperature^33,35^. The superposition of data from the two muscle fibres (Fig. 4c, soleus: circles, pooled data from Fig. 4a; psoas: reverse triangles, pooled data from ref. ^33^ and new experiments) provides striking further elements in support of the finding that cooperativity depends on the motor force allowing the identification of the underlying molecular mechanism. The first element is that the *n*_H_-*F*_0_ relation of fast muscle appears as the continuation, in the higher *F*_0_ range, of the same relation as that of slow muscle, indicating that the mechanism encompasses the muscle type, relating *n*_H_ to the motor force per se. Notably, the mean *n*_H_-*F*_0_ point of the soleus fibre at 35 °C is not significantly different from the corresponding point of the psoas fibre at 6 °C (Table 1), further solidifying the idea that the only determinant of *n*_H_ is *F*_0_, without any further effect of temperature or protein isoform. As a corollary it can be seen that the corresponding *n*_H_ − *T*_0_ points belong to different relations depending on the myosin isoform (Supplementary Fig. 6, circles soleus, reverse triangles psoas). The psoas *n*_H_ − *T*_0_ point at 6 °C is leftward shifted with respect to the soleus *n*_H_ − *T*_0_ point at 35 °C, with which it shares the same *F*_0,_ because of the isoform-dependent effect on *T*_0_^35^. In fact, the isometric duty ratio is higher in soleus than in psoas and when the comparison is made at the respective temperature at which *F*_0_ is the same, soleus *T*_0_ is higher than psoas *T*_0_ (Table 1).

The second element is that *F*_0_ of fast muscle attains values at which the cooperative mechanism shows a saturation kinetics that suggests a Michaelis-Menten reaction. Indeed, when *n*_H_-*F*_0_ data are plotted as reciprocals (Fig. 4d), the 1/*n*_H_−1/*F*_0_ relation can be fitted by a first order equation (continuous line) that, according to the Michaelis-Menten kinetics, gives an ordinate intercept that estimates 1/*n*_H,max_ (the reciprocal of the maximum value of *n*_H_ attained at saturating values of *F*_0_) and an abscissa intercept that estimates -1/*K*_M_ (the negative reciprocal of *K*_M_, the value of *F*_0_ at which *n*_H_ is half-maximum). *n*_H,max_ and *K*_M_ are 8.34 ± 1.41 and 5.12 ± 0.95 pN respectively. Notably *K*_M_ is larger than the maximum *F*_0_ achieved in soleus fibres, which explains the apparent linearity of the *n*_H_-*F*_0_ relation in this muscle with the evidence that its occupancy of the whole *n*_H_-*F*_0_ relation is limited to the lower branch of the hyperbola.

Increase in temperature also induces increase in pCa_50_ (circles in Fig. 4b; see also refs. ^24,39–41^). Obviously, whenever the *n*_H_-*F*_0_ points belong to force-pCa relations that share the same threshold, as it occurs when the force-pCa is modulated by temperature (Fig. 2b and ref. ^24^), an increase in *n*_H_ necessarily underpins an increase in pCa_50_. The correlation between *n*_H_ and pCa_50_ is lost when extended to different fibre types, as demonstrated by the finding that the same force-pCa relations of the psoas fibres holding the highest *n*_H_ values in Fig. 4c (reverse triangles) and Table 1 hold the smallest pCa_50_ values in Table 1. This can be explained considering that the Ca^2+^-sensitivity of the thin filament, and thus the threshold pCa and pCa_50_ depend primarily on the TnC isoform, while cooperativity does not: the slow skeletal/cardiac TnC has higher Ca^2+^ affinity and larger pCa_50_ than the fast skeletal TnC^42–44^, but lower cooperativity as expected by its dependence on *F*_0_.

### Considerations in relation to previous work

The association of the rightward shift of the force-pCa relation with the increase in *n*_H_ in fast fibres with respect to slow fibres could indicate a relation between reduction in Ca^2+^ sensitivity and increase in cooperativity. In this respect, however, it must be noted that there is a systematic association between the isoforms of TnC and HMM: fast muscle fibres with fast MHC express the fast TnC, while slow muscle fibres with the slow MHC isoform express the slow TnC isoform^45^. The larger *n*_H_ of the fast muscle fibres can be explained by the presence of the fast MHC isoform, which develops a higher *F*_0_, not dependent on the TnC isoform. The co- presence of the fast TnC isoform explains per se the rightward shift of the force-pCa relation. This conclusion agrees with previous works^26,27^ showing that, in fast skeletal muscle fibres, the replacement of native TnC with TnC mutated for its affinity for Ca^2+^ produced shift of the pCa_50_ to either the left (when the Ca^2+^ affinity was increased) or the right (when the Ca^2+^ affinity was reduced) without significant effect on the cooperativity. The above conclusion is challenged by results of experiments on fast skeletal muscle myofibrils in which the fast isoform of the Tn complex was replaced with the slow/cardiac isoform^46^. The substitution produced a leftward shift of pCa_50_ accompanied by a marked reduction of *n*_H_ from 3.3 to 1.4, that is a value even smaller than that found in general in cardiac myocytes or myofibrils (∼3^47^,). Notably the replacement of the fast TnC isoform with the slow/cardiac isoform did not affect significantly either pCa_50_ or *n*_H_, even if it reduced the mechanical performance (maximum isometric force, rate of force development). That the issue for the slow/cardiac muscle is more complicated than for the fast skeletal muscle is demonstrated by the finding that in cardiac myofibrils, in contrast to the finding in fast skeletal myofibrils^26,27^, the replacement of native cardiac TnC (cTnC) with cTnC mutated for its affinity for Ca^2+^ produced shift of the pCa_50_ to either the left (when the Ca^2+^ affinity was increased) or the right (when the Ca^2+^ affinity was reduced), in both cases accompanied by the reduction of *n*_H_^48^. This peculiar response of cTnC substitution in cardiac myofibrils could be related to the reported evidence that in cardiac muscle motor attachment more strongly affects cooperativity increasing either Ca^2+^ binding to cTnC per se or the action of cTnC in the regulatory complex^49–51^. The finding in this work that cooperativity depends on the force of the motor suggests that a substantial contribution to solve the question would be to apply the half-sarcomere compliance analysis shown in this paper to test whether and how the replacement of the regulatory proteins changes the force of the myosin motor.

Previous works showed that the slope of the force-pCa relation at forces below the midpoint is higher than that at forces above the midpoint^24,52,53^. This asymmetry is expected if at low [Ca^2+^] more than one mechanism, as for instance the recently discovered mechanosensing-based myosin filament activation^6^, contributed to cooperativity. In fact, while the thin filament activation is complete only with Ca^2+^ saturation at the maximum force (*T*_0,4.5_), mechanosensing-based myosin filament activation is already complete when the force is nearly half-maximum^8^. Thus, where present, the effect of partial myosin filament activation could affect the shape of the first half of the force-pCa relation, making the whole relation asymmetric across the force midpoint and weakening the solidity of our analysis that is based on the explanation of the slope of the relation only with the thin filament cooperative activation. In this respect, a crucial finding in our work is that, independently of temperature, the force-pCa relation for the soleus fibre is symmetric across the force midpoint (Fig. 2b, filled circles 12 °C, open circles 35 °C). A more stringent test for the presence of asymmetry is obtained with the linearization of the force-pCa relation by expressing the force as log [*T*_0_/*T*_0,4.5_/(1 – *T*_0_/*T*_0,4.5_)], The linear plots do not show change in the slope across the zero ordinate, corresponding to the force midpoint (Supplementary Fig. 5, filled circles 12 °C, open circles 35 °C), as it is expected from a symmetric force-pCa relation. Considering the previous finding in our lab that also the psoas fibre exhibits a symmetric force-pCa relation^54^, we can conclude that in both muscle types there is only one cooperativity mechanism throughout the whole pCa range^38^.

The absence in our experiments of a significant effect of myosin filament activation on the lower half of the force-pCa relation is likely due to the use of the temperature jump technique for eliciting the contraction. The method is essential to avoid the sarcomere disorder caused by the time for Ca^2+^ equilibration within the fibre and preserve the possibility to apply fast mechanical methods to a selected population of sarcomeres^33^. As demonstrated in ref. ^14^, the myosin filament structure of relaxed skinned fibres has already undergone a substantial transition to the ON/disordered state at 10 °C and presumably this transition is even more effective at 1 °C.

In works of other laboratories, in which contractions are elicited by directly shifting the fibre from low- to high- [Ca^2+^] solution, the time needed for [Ca^2+^] equilibration within the fibre implies that force development is accompanied by increase in sarcomere disordering. This per se can provide an explanation for the reported asymmetry in the force-pCa relation since sarcomere disordering is larger at larger forces (smaller pCa) and thus more effectively depresses the upper half of the force-pCa relation. Moreover, the consequences of Ca^2+^ diffusion time are likely worse in fast fibres versus slow fibres, due to the faster rate of force development, which makes more effective the delay in [Ca^2+^] equilibration. This would provide an explanation for the finding that the maximum isometric force (*T*_0,4.5_) has been reported to be similar in slow and fast fibres^24^, while in our experiments it is 70–90% higher in fast than in slow fibres (Table 1, but see also ref. ^35^).

Our high-resolution sarcomere-level mechanics allowing in situ measurement of the force per motor provides a key for explaining the apparent contradiction that changes in *T*_0_ are found both related and unrelated with *n*_H_. We demonstrate that, when changes in *T*_0_ of a given muscle type are completely explained by changes in *F*_0_ (as it occurs by changing temperature in soleus fibres), the same relation to *n*_H_ holds for either *F*_0_ or *T*_0_ (Supplementary Fig. 6, open and filled circles). On the contrary, other interventions that induce depression in *T*_0_ as the raise in orthophosphate (Pi) or vanadate (Vi) concentrations have been found not to affect significantly the cooperativity parameter of the force-pCa relation^24,55^. According to the present work, those results find a straightforward explanation in the demonstration that the reduction of *T*_0_ obtained by increasing either Pi^56^ or Vi^57^ is due to a proportional reduction in the number of attached motors, without effect on *F*_0_.

### Revision of the classical steric-blocking model and the effect of OM on cooperativity

The *n*_H_-*F*_0_ relation determined in our experiments demonstrates that the classical two step steric-blocking model^21^ must be implemented with the introduction of the concept of the dynamic nature of the movement of Tm promoted by motor attachment: the amplitude of the azimuthal movement of Tm and its axial extension, which should represent the intermediate step in terms of Michaelis-Menten kinetics, depend on *F*_0_ with a saturation kinetics and so does the number of cooperatively activated actin monomers (the final product of the reaction).

The intermediate reaction hypothesised above assumes the presence of a link between force development and azimuthal movement of Tm, which may find support in mechanical and structural evidence that the temperature-dependent increase in the isometric force is related to a higher free energy change between different conformations of the attached myosin motors that shifts the equilibrium to higher force generating states^31,32,58,59^ and likely implies an azimuthal reorientation of the motor on the actin monomer it is bound to^60,61^. Further structural evidence for an azimuthal component of the working stroke that promotes Tm displacement can be found in cryo-EM images of isometrically contracting Insect Flight Muscle^62^. Alternatively, it could be argued that it is the Tm that modulates the generation of force by myosin in relation to either temperature or its slow and fast isoform expression in slow and fast muscle fibres respectively.

Addition of OM induces a drastic reduction in *n*_H_ and in the *n*_H_-dependence on average *F*_0_, which cannot be accounted for by the reduced value of *F*_0_ (Fig. 4a, triangles). The explanation resides in the specific mechanism by which the average *F*_0_ is reduced by OM: while reduction of the average *F*_0_ by reducing temperature is accounted for by shifting of the whole population of attached motors towards lower force generating states of the working stroke^31^, the reduction of the average *F*_0_ by OM is attributed to arresting the force generating mechanism in OM-motors^29^. In this way an OM-motor would counteract the force-dependent Tm displacement by a neighbour force-generating motor, causing a direct inhibition of cooperativity.

The mechanisms underlying the dynamic nature of the action of the myosin motor on thin filament cooperative activation and the dual effect of OM (increase in Ca^2+^ sensitivity and inhibition of cooperativity) are qualitatively tested with a simplified representation of the sequence of the events occurring just beyond the threshold for Ca^2+^ activation (Fig. 5), providing fundamental constraints for the implementation of a quantitative model able to give a detailed description of the role of the motor force on the dynamics of thin filament activation and for the definition of the effects of small molecules as possible therapeutic tools.

## Methods

### Animals and ethical approval

Experiments have been done on demembranated fibres from the soleus muscle of adult male New Zealand white rabbits at the PhysioLab Research Unity of the Biology Department of the University of Florence. Some additional experiments, beyond those already published in ref. ^33^, were done also on demembranated fibres from the psoas muscle of the rabbit. The experiments were carried out according to the protocols approved by the Ethical Committee of the University of Florence and by the Italian Ministry of Health (authorization n. 956/2015 PR) in compliance with the Italian regulation on animal experimentation, Decreto Legislativo 26/2014 and the EU regulation (directive 2010/63) (authorization n. 55FF7.N.THO). Rabbits (4–5 kg weight, 20–30 weeks old) were sacrificed by injection of an overdose of sodium pentobarbitone (150 mg kg^-1^) in the marginal ear vein. Three rabbits were used for this work. All animals have been kept with free access to food and water prior to use.

### Fibre preparation and mechanical apparatus

Small bundles (50–100 fibres) dissected from soleus muscle were stored in skinning solution containing 50% glycerol at −20 °C for 3–4 weeks and single fibres were prepared just before the experiment as already described^33,63^. The same procedure was followed in the few additional experiments made on the psoas fibres of the rabbit. The increase of interfilamentary distance following cell membrane permeabilization was reversed by the addition of the osmotic agent Dextran T-500 (4% weight/volume)^33,64–68^. A fibre segment 4–6 mm long was clamped at its extremities by T-clips and mounted between the lever arms of a loudspeaker motor, able to impose steps in length complete within 100 μs^69^, and a capacitance force transducer, with resonant frequency 40–50 kHz^70^. The extremities of the fibre were fixed first with a rigor solution containing glutaraldehyde (5% v/v) and then glued to the clips with shellac dissolved in ethanol (8.3% w/v^68,71^). This procedure prevents the sliding of the ends of the fibre segment inside the clips and minimizes the shortening of the activated fibre against the damaged sarcomeres at the ends of the segment during force development. Sarcomere length (*sl*), width (*w*) and height (*h*) of the fibre were measured at 0.5 mm intervals in the 3–4 mm central segment of the relaxed fibre with a 40x dry objective (Zeiss, NA 0.60) and a 25x eyepiece. The fibre length (*L*_0_) was adjusted to have a *sl* of 2.3–2.5 µm. The fibre cross-sectional area (CSA) was determined assuming the fibre cross-section as elliptical (CSA = *π*/4·*w*·*h*) and its value, in the presence of 4% dextran, ranged between 1900 and 5800 µm^2^ (3500 ± 1000 µm^2^, mean ± SD from 37 fibres). Fibres were activated by temperature jump using a solution exchange system^33^. A striation follower^72^ allowed nanometer-microsecond resolution recording of length changes in a selected population of sarcomeres (range 500–1200 sarcomeres) starting at the time the optic path was permitted through the glass window in the floor of the test temperature drop (see^33^ for details). Data in control are from 31 fibres out of 37 fibres, data in the presence of 1 µM OM from 19 out of 37 fibres. The effect of temperature on the relevant mechanical parameters (Fig. 2 and Fig. 3) has been determined at 12, 17, 20, 25, 30 and 35 °C in control (31 fibres) and at 12, 20, 25, 30 and 35 °C in the presence of 1 µM OM (17 fibres). The force-pCa relations have been determined at 12, 17, 25 and 35 °C in control (19 fibres) and at 12, 25 and 35 °C in the presence of 1 µM OM (11 fibres) (Table 1).

### Force-pCa relation

The dependence of active isometric force on [Ca^2+^] in demembranated fibres from soleus muscle was estimated in the range of pCa values from 8 to 4.2. The force-pCa relation was interpolated by the Hill equation:^73^

1 $${T}_{0}/{T}_{0,4.5}=1/[1+{10}^{{n}_{{{{{{\rm{H}}}}}}}\cdot (pCa-pC{a}_{50})}]$$

where *T*_0,4.5_ is the isometric force at saturating [Ca^2+^] (pCa ∼4.5), *n*_H_, the Hill coefficient, indicates the slope of the relation and thus the degree of cooperativity of thin filament activation, pCa_50_, the pCa value for *T*_0_ = 0.5·*T*_0,4.5_, is an estimate of the Ca^2+^ sensitivity of the contractile system. The force-pCa relations were determined at different temperatures (range 12–35 °C) in a sequence varied at random in the absence (control) and in the presence of 1 μM OM.

### Stiffness measurements and estimate of the contributions of myofilaments and myosin motors to half-sarcomere compliance

Step length changes (ranging from −2 to +2 nm per hs, stretch positive), complete in 110 μs, were imposed on the isometrically contracting fibre (Supplementary Fig. 2a–c) to estimate the half-sarcomere stiffness (*k*_0_). To enhance the precision of the measurement, a train of different-sized steps at 200 ms intervals was superimposed on *T*_0_ and, to maintain the sarcomere length and *T*_0_ constant before the test step, each test step was followed, after a 50-ms pause, by a step of the same size but in opposite direction. *k*_0_ is measured by the slope of the relation between the force attained at the end of the step (*T*_1_) and the change in half-sarcomere (hs) length recorded by the striation follower^72^ in a segment of the fibre (*T*_1_ relation, Supplementary Fig. 2d) (see also ref. ^68^).

The stiffness of the array of motors in the half-sarcomere (*e*_0_) was calculated from *k*_0_ using a simplified mechanical model of the half-sarcomere (Model 1, Supplementary Fig. 1), in which the hs compliance (*C*_hs_ = 1/*k*_0_) is defined by the sum of the compliance of the array of motors (1/*e*_0_, the reciprocal of the stiffness) and the cumulative equivalent compliance of the actin and myosin filaments (*C*_f_):

2 $${C}_{hs}=(1/{k}_{0})={C}_{f}+1/{e}_{0}$$

Under conditions in which *T*_0_ changes in proportion to the number of motors, like in isometric contractions at different pCa^33^, the strain of each motor, *s*_0_, remains constant and the compliance of the motor array changes in inverse proportion to *T*_0_: 1/*e*_0_ = *s*_0_/*T*_0_ and thus *s*_0_ = *T*_0_/*e*_0_. The strain of the half-sarcomere (*Y*_0_ = *C*_hs_*T_0_) can be expressed by the sum of filament strain (*C*_f_·*T*_0_) and the motor strain. Thus, from Eq. 2:

3 $${Y}_{0}=({C}_{hs}\cdot T_{0}={C}_{f}\cdot {T}_{0}+{T}_{0}/e_{0}=){C}_{f}\cdot {T}_{0}+{s}_{0}$$

In this case (i) the slope and the ordinate intercept of the first order equation fit to the half-sarcomere strain-force (*Y*_0_ − *T*_0_) relation (Supplementary Fig. 3b) estimate *C*_f_ and *s*_0_ respectively and (ii) the stiffness of the array of myosin motors (*e*_0_) increases in proportion to the Ca^2+^-dependent increase in *T*_0_ (Supplementary Fig. 3c), underpinning a proportional increase in the fraction of attached motors.

For *T*_0_ < 50 kPa the *Y*_0_ − *T*_0_ data are shifted downward with respect to the linear fit to data ≥50 kPa (continuous line in Supplementary Fig. 3b). This deviation from linearity at very low forces has been explained with the presence of an elastic element in parallel with the myosin motors (Model 2 in Supplementary Fig. 1) with a compliance *C*_p_ that is one order of magnitude larger than the compliance of the array of motors at saturating [Ca^2+^]. Consequently, the *Y*_0_-*T*_0_ relation is affected only in the region where the number of the motors (and thus their cumulative stiffness) is so low to become comparable to the stiffness of the parallel elastic element^35,74^. The contribution of this parallel elasticity emerges also as an upward deviation of the *e*_0_ − *T*_0_ relation for forces <50 kPa (Supplementary Fig. 3c). In this respect it has been demonstrated that the first order equation fit to data for forces ≥50 kPa (continuous lines in Supplementary Fig. 3b, c) provides estimates of *s*_0_, *C*_f_ and *e*_0_ that are not significantly affected by the parallel elasticity^35,74^. Notably, the *Y*_0_ − *T*_0_ relation in 1 µM OM (Supplementary Fig. 3d) does not show deviation from linearity for the same low forces at which the control relation shows the downward shift, because in this case the number of attached motors and thus their cumulative stiffness are higher than in control^30^.

### Data collection and analysis

Force, motor position and sarcomere length signals were recorded with a multifunction I/O board (PXIe-6358, National Instruments). A program written in LabVIEW (National Instrument) was used for signal generation and data acquisition. All data were analysed using dedicated programs written in LabVIEW (National Instruments) and Microsoft Excel and Origin 2018 (OriginLab Corp., Northampton, MA, USA) software.

### Statistics and reproducibility

For the slow and fast fibres in the control and for the slow fibres in the presence of 1 μM OM the statistical significance of the temperature effect is tested with one-way ANOVA. Error bars on mean data points are ± SEM.

### Solutions

The composition of the solutions (Supplementary Table 1) was calculated with a computer program similar to that described in ref. ^75^ and^63^ by taking into account the effect of temperature on the equilibrium between two ions (Ca^2+^ and Mg^2+^) and two chelators (EGTA and ATP) by using the free available Maxchelator software (https://somapp.ucdmc.ucdavis.edu/pharmacology/bers/maxchelator/CaMgATPEGTA-NIST-Plot.htm) developed by Dr. Chris Patton (see also^76^). Due to the temperature dependence of the pK of the buffer used, the total concentration of TES decreases with the increase in temperature from 140 mM (at 5 °C) to 40 mM (at 35 °C)^77^. Free Mg^2+^ and MgATP were in the range 1.7–1.9 mM and 4.9-5.0 mM respectively, with 190 mM ionic strength. Cysteine and cysteine/serine protease inhibitors (trans-epoxysuccinil–L–leucylamido–(4–guanidine) butane, E–64, 10 µM; leupeptin, 20 µg/ml) were also added to all solutions, in order to preserve lattice proteins and thus sarcomere homogeneity. The activating solution at a given pCa (range 8-4.2) was obtained by mixing relaxing and activating solution. OM (CK-1827452, Selleckchem) was dissolved in DMSO (Sigma D-5879) to obtain a 17.5 mM stock solution. OM concentration in the final solutions (1 μM) was obtained by dilution starting from the stock solution^30^. 4% dextran T-500 (Thermo Fisher Scientific) was added to all solutions to restore the lattice spacing before skinning^33,35,64,65^.

### Reporting summary

Further information on research design is available in the Nature Research Reporting Summary linked to this article.

## Supplementary information

Peer Review File

Supplementary Information-New

Description of Additional Supplementary Files

Supplementary Data 1

Reporting summary

## Data Availability

The authors declare that the data supporting the findings of this study are available within the paper and its Supplementary Information files. The source data for Figs. 2, 3, 4, Supplementary Figs. 2, 3, 4, 5 and 6 are provided as a Supplementary Data 1 excel file. All remaining data will be available from the corresponding author upon reasonable request.
