# Supplementary material for: The force of the myosin motor sets cooperativity in thin filament activation of skeletal muscles
Source: Commun Biol. 2022 Nov 18;5:1266. doi: 10.1038/s42003-022-04184-0 (PMC9674696; doi:10.1038/s42003-022-04184-0)
Supplement: Supplementary file 2 — Supplementary Information-New [file 42003_2022_4184_MOESM2_ESM.pdf]

Supplementary Information for

The force of the myosin motor sets cooperativity in thin filament activation of skeletal muscles

Marco Caremani[1,2], Matteo Marcello[1,2], Ilaria Morotti[1,2], Irene Pertici[1,2], Caterina Squarci[1,2], Massimo Reconditi[1,3], Pasquale Bianco[1,2], Gabriella Piazzesi[1,2], Vincenzo Lombardi[1,*] and Marco Linari[1,2]

[1]PhysioLab, University of Florence, Florence, Italy, [2]Department of Biology, University of Florence, Florence, Italy, [3]Department of Experimental and Clinical Medicine, University of Florence, Florence, Italy

[*]Corresponding author: Vincenzo Lombardi.
Email: vincenzo.lombardi@unifi.it

This PDF file includes:

Supplementary Figure 1

Supplementary Figure 2

Supplementary Figure 3

Supplementary Figure 4

Supplementary Figure 5

Supplementary Figure 6

Supplementary Note 1

Supplementary Table 1

Supplementary Table 2

Supplementary References

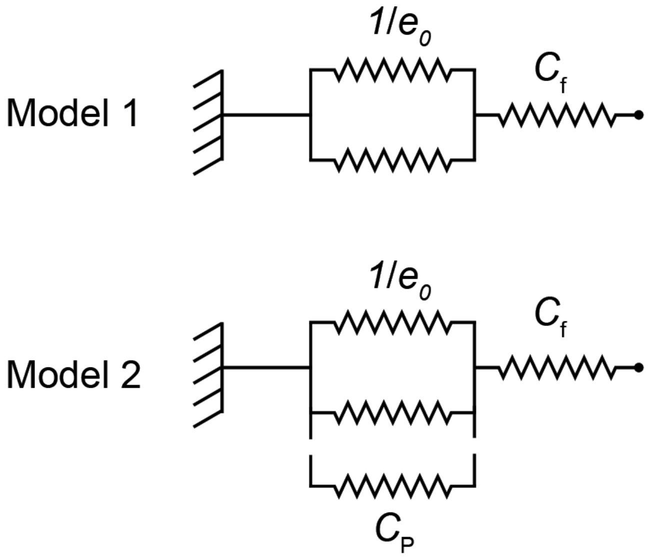

Supplementary Figure 1. Models representing the elements that contribute to the half-sarcomere compliance. In model 1, the myofilament compliance (C_f) is in series with an array of in parallel elastic elements, representing the attached myosin motors (only two are shown for simplicity) with a compliance $1/e_0$, where e_0 is the equivalent stiffness of the array. In fibres activated at different [Ca^{2+}], the isometric force exerted by the array increases linearly with the number of attached motors, so that the stiffness of the array is proportional to the isometric force. In model 2, an elastic element with compliance C_P (stiffness $1/C_P$) independent of the isometric force, is added in parallel with the array of motors.

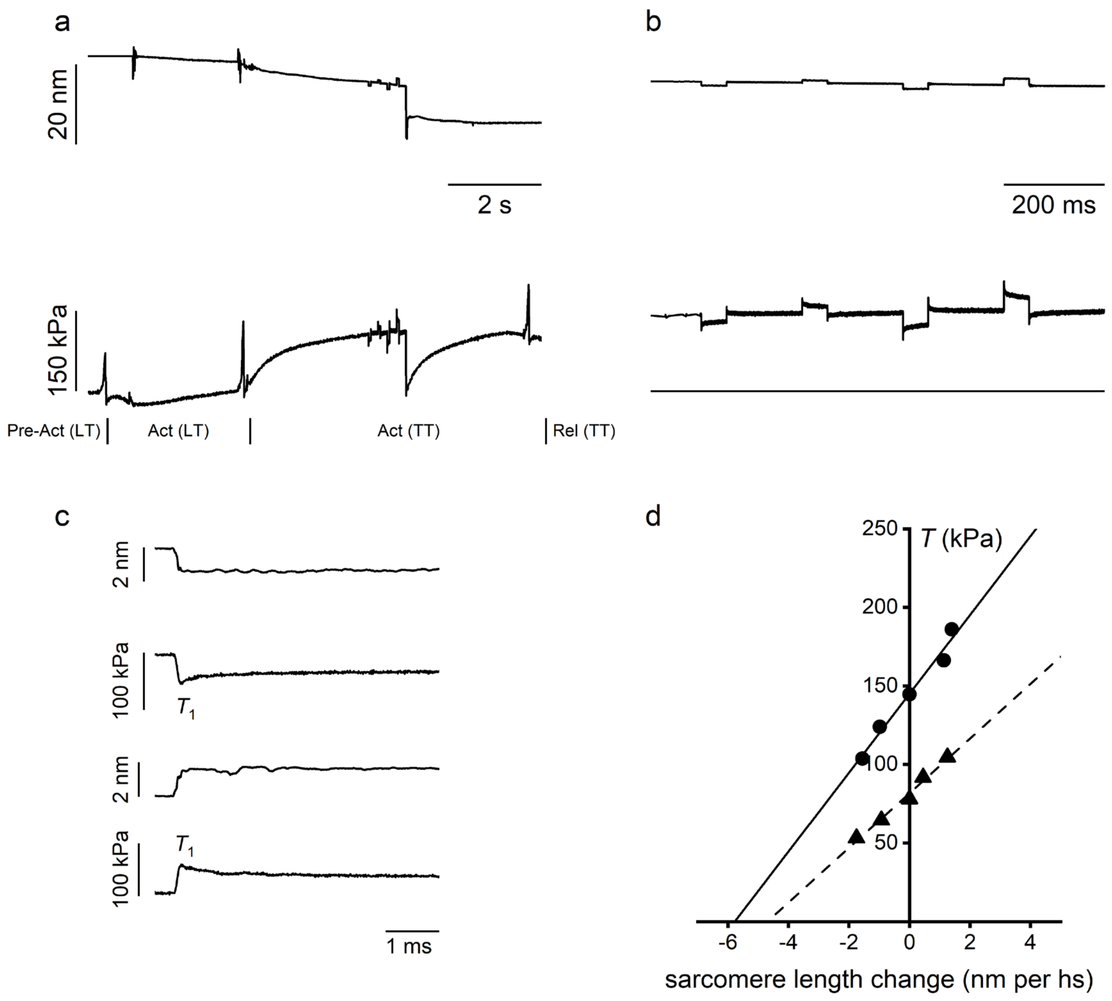

Supplementary Figure 2. Measure of the half-sarcomere stiffness. a. Mechanical protocol consisting in a train of step length changes imposed on the fibre at the isometric force attained following a temperature jump from the low temperature drop (LT, 1.7 °C) to the test temperature drop (TT, 12.2 °C in this case). Pre-Act, pre-activating solution; Act, activating solution (pCa 4.5 in this case); Rel, relaxing solution. Vertical bars below the force trace indicate the transition to the next drop. Upper trace, length change per hs; middle trace, force response; lower trace, force baseline. **b.** Traces in a on a faster time scale with the same vertical calibration. **c.** Force responses to the small step release (upper panel) and stretch (lower panel) on an even faster time scale to show T_1, the tension attained at the end of the step. In each panel: upper trace, hs length change; lower trace, force. **d.** T_1 relations from the same experiment as in a-c upon activation at pCa 4.5 (circles) and 6.4 (triangles). Lines are the first order regression equation fitted to the data. Fibre length, 5.3 mm; segment length under the striation follower, 0.93 mm, average segment sarcomere length, 2.45 μm, CSA, 2800 μm^2.

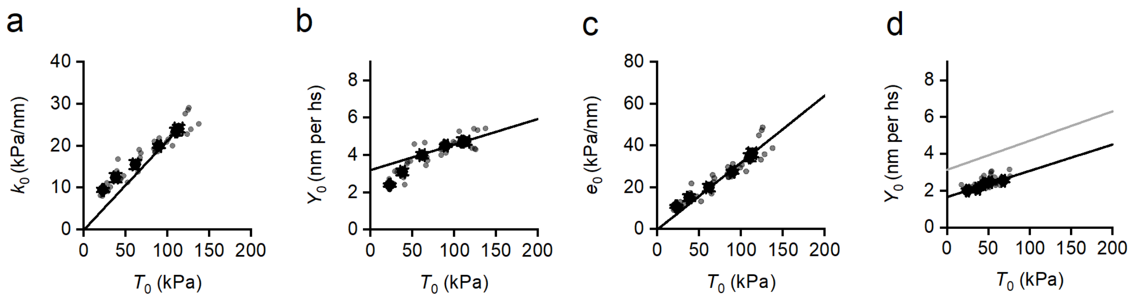

Supplementary Figure 3. Dependence of the relevant mechanical parameters of the half-sarcomere on Ca^{2+}-dependent T_0. Temperature 12 °C. **a**. hs stiffness k_0, in control. The line indicates direct proportionality between k_0 and Ca^{2+}-dependent T_0. **b**. hs strain Y_0, in control. The line is the first order regression equation fitted to the pooled data for force values ≥50 kPa (Eq. 3 in Methods). **c**. Stiffness of the array of attached motors e_0, in control. The line is the first order regression equation fitted to pooled data for force values ≥50 kPa. The slope is 0.32 ± 0.03 nm^{-1}; the ordinate intercept, -0.13 ± 2.97 kPa/nm, is not significantly different from zero (P>0.95). **d**. Same relation as in b in the presence of 1 µM OM. Black line is the first order regression equation fitted to the pooled data. The grey line is the black line as in b for comparison. Grey circles pooled data and black circles mean ± SEM from eight fibres in a-c and five fibres in d. pCa values corresponding to T_0 from left to right are 6.77, 6.59, 6.38, 6.05, 5.77 and 4.50 in a-c and 7.42, 7.04, 6.82, 6.45 and 4.50 in d.

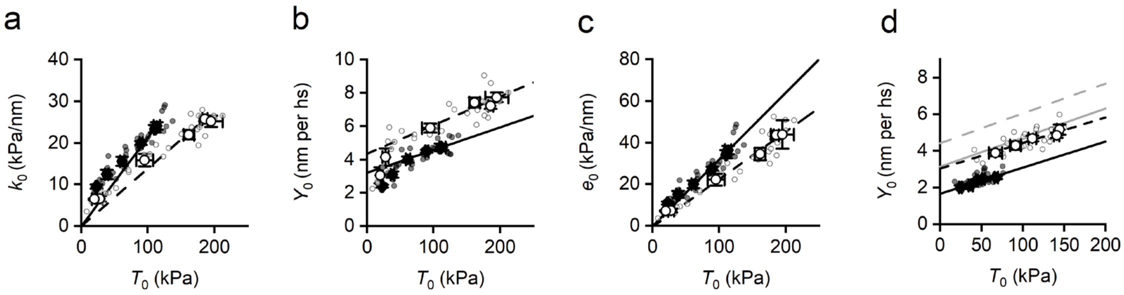

Supplementary Figure 4. Effect of rise in temperature to 35 °C on the same relations as those in Supplementary Figure 3. Open symbols and dashed lines 35 °C (four fibres); filled circles and continuous lines 12 °C (from Supplementary Figure 3). Panels follow the same definitions as in Supplementary Figure 3. In c the slope and ordinate intercept of dashed line fitted to pooled data (grey open circles) are 0.23 ± 0.02 nm^{-1} and -0.50 ± 3.53 kPa/nm (for the ordinate intercept P > 0.85). pCa values of T_0 at 35 °C from left to right are 7.22, 6.98, 6.74, 6.37, 6.00 and 4.50 in a-c and 7.17, 6.92, 6.60, 6.00 and 4.50 in d.

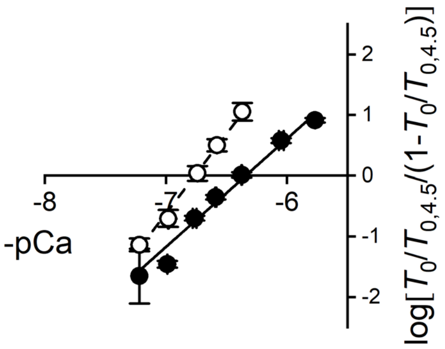

Supplementary Figure 5. Linearised force pCa-relations. Data from Figure 2b; filled circles and continuous line, data and first order equation fit at 12 °C; open circles and dashed line, 35 °C. Fit parameters: continuous line, slope 1.78 ± 0.13 and ordinate intercept 11.31 ± 0.85; dashed line, slope 2.65 ± 0.13 and ordinate intercept 17.92 ± 0.85. The slope at either temperature is not significantly different from the corresponding n_H determined with Hill's fit in Figure 2b and reported in Table 1 (P always >0.05).

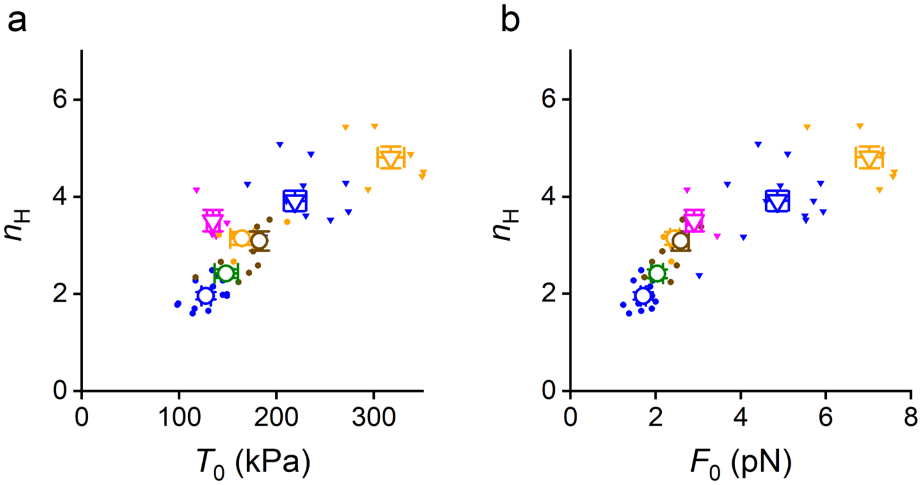

Supplementary Figure 6. Dependence of n_H on T_0 (a) and F_0 (b) modulated by temperature in control solution. Superimposed relations from the soleus fibres (circles) and from psoas fibres (reverse triangles). Same data as in Fig. 4c. Different colours refer to different temperatures as reported in Figure 4 (6 °C, magenta; 12 °C, blue; 17°C, green; 25 °C, orange; 35 °C brown). Filled symbols pooled data and open symbols means ± SEM.

Supplementary Note 1

To keep the model as simple as possible we limit the description of the events at [Ca^{2+}] just above the threshold of the force-pCa relation, at which only a few RU's are activated by Ca^{2+}. When the 3D lattice of thin and thick filaments at full filament overlap is reduced to one thick filament facing a single stranded thin filament, the number of myosin dimers per RU can be assumed to be 2, according to the calculation here following. A single RU is composed of 7 actin monomers (5.5 nm in diameter each), 1 Tn complex and 1 Tm (Figure 1) and repeats every (5.5 nm * 7 monomers=) 38.5 nm, so that there are about 26 RU's per strand in the 1000 nm long thin filament [1], and 52 RU's for the double stranded thin filament. At full filament overlap, in the overlap zone (700 nm long) there are (52*700/1000 =) 36 RU's able to interact with myosin. Thick filament is 1600 nm long and, considering the bare zone 200 nm, the axial distance between consecutive crowns of myosin motors 14.5 nm [2,3] and 3 myosin molecules per crown, there are (700 nm/14.5 nm=) 49 crowns [4] and (49*3=) 147 myosin molecules per half-thick filament (htf). In the same overlap zone there are (700 nm/38.5 nm * 14=) 250 actin monomers per double stranded thin filament available for interaction with myosin. Considering the ratio 2:1 of thin over thick filament the total number of actin monomers rises to 500, thus the ratio of myosin molecules per actin monomer is (147/500 =) 0.29, which in our representation gives (0.29*7 =) 2 myosin molecules available for binding actin in each RU.

For testing the mechanism underlying F_0-dependence of cooperativity in control (Figure 5 a and b) we consider a minimum representative number of RU's = 12 of which, at just above threshold (pCa= 7), 10 are blocked (actin monomers, white circles) and 2 have undergone the blocked-closed transition by binding of Ca^{2+} (yellow) to Tn (blue) that displaces Tm (black line) upward making actin monomers available for strong motor attachment (cyan circles). In the thick filament (grey) there are two myosin molecules (dimers) per RU, but only 1 motor per dimer (red, 24 altogether) is shown for clarity, as at physiological ATP only one motor at a time interacts with actin. 6 of the 24 motors emerge from the backbone to indicate that they are available for actin binding, while the others lie on the surface of the thick filament to indicate that they are unavailable, either because in their OFF state or for steric and biochemical reasons. A unique graphical representation of motors unable to attach to actin is given for simplicity. Thus, the fraction of motors able to attach to an activated actin monomer in control is (6/24 =) ¼. This is justified by the assumption that, under the condition of the present experiments (temperature jump activation), most of myosin motors are already in the disordered ON state, able to attach to actin following Tm displacement caused by either Ca^{2+}-activation of the RU or the cooperative action of a nearby attached motor. Under this condition the attachable fraction in control (1/4) is just below the duty ratio of the isometric contraction (classically ~1/3).

The basic assumptions concerning the cooperative mechanism are: (*i*) given the head-tail interaction between Tm of consecutive RU's, attachment of a motor can displace Tm and spread

the availability of actin sites also beyond the corresponding RU; (*ii*) longitudinal extent of Tm displacement and number of neighbouring actin monomers (n_A) made available on either side of the monomer strongly bound to a motor depend on motor force F, which corresponds to the average motor force F_0 determined experimentally in control (Table 1) and, at a given temperature, is the same for all force generating motors, independent of the presence/absence of OM in the solution. n_A depends on F according to the expression $n_A = 4 + 2.6 * F$ At 12°C (F = 1.7 pN) $n_A \sim 8$ and at 35°C (F= 2.6 pN) $n_A \sim 11$; (*iii*) OM-motors have $F = 0$ and thus, following the attachment of an OM-motor, $n_A = 4$. If the actin monomer to which the OM motor attaches is involved in the cooperative action of other attached motors, the action of the other motors is inhibited limiting Tm displacement and n_A according to the action of the OM-motor.
In Figure 5, panels a (12 °C) and b (35 °C) show the sequence of events in control that explains how the cooperativity depends on temperature-modulated value of F. The pCa threshold for triggering the first motor attachment is set just below 7 independently of temperature (Figure 2a and Ref [7]); the axial positions of the two Ca^{2+} activated RUs (3 and 9) and of the six available motors (6, 7, 10, 14, 19 and 23) are kept constant at either temperature as shown in line 1. Motor 6 can attach, generate force and spread thin filament activation in either direction, by displacement of Tm through 8 actin monomers at 12 °C (line 2 in a) and 11 monomers at 35 °C (line 2 in a). Consequently, motor 7 can attach, generate force and further spread thin filament activation through 8 actin monomers at 12 °C (line 3 in a) and 11 monomers at 35 °C (line 3 in b). At 12 °C there are no ON motors in correspondence of the newly activated monomers, so the process stops and can proceed further only if an increase in [Ca^{2+}] activates other RU's. At 35 °C, instead, motor 10 can attach, generate force and further spread thin filament activation (line 4 in b). No other motors are ON in correspondence of the newly activated monomers and the process stops. The outcome of the example is that the rise in [Ca^{2+}] just above the threshold that activates 2 RU's, triggers the development of a force of 2*F at 12°C and 3*F at 35°C, verifying the mechanism that makes the degree of cooperativity of thin filament activation to depend on F_0.

Panels c and d of Figure 5 show the sequence of events (at 35°C) that explains the double action of OM, namely the leftward shift of the threshold of the force-pCa relation with increase in pCa_{50} and the drop in n_H. For simplicity we assume, from the known stoichiometry of OM binding to motors at 12 °C [8], that also at 35 °C in 1 µM OM 50% of the motors have OM in their specific site, that is 12 are OM-motors (brown) and 12 motors (red) (line 1 in c-d). Moreover, the number of available motors is reduced to 3 (6, 10 and 19) to keep their fraction (1/4) the same as in a-b, while the number of available OM-motors (that must be higher taking into account the activating effect of OM at low Ca^{2+} [9,10]), is set to 5. Three are in position 7,14 and 23 (the same positions as ON motors in control) and two in new positions, 2 and 18 (line 1 in c-d). Thus, the fraction of motors available for attachment to actin in OM is (8/24 =) 1/3. Given the increase in spatial frequency of all available

motors (both with and without OM bound), the pCa for threshold events is shifted leftward at a pCa (just above 7.7) at which only one RU (9) is Ca^{2+} activated (panel c line 1, a condition that would be subthreshold in control). OM-motor 18 can attach and, since F=0, spread thin filament activation on either direction only through 4 actin monomers (line 2 in c). This is enough for motor 19 to attach, generate force and further spread activation through 11 monomers (row 3 in c). In the absence of ON motors in correspondence of the newly activated monomers, the process stops and can proceed further only if an increase in [Ca^{2+}] activates other RU's. Panel d is representative of the events at pCa 7, a sub-saturating pCa at which the observed force-pCa relation in OM approaches that in control (Fig. 3b). Two RU's are activated at this pCa (3 and 9, panel d line 1), in agreement with line 1 in panels a-b. Together with the events triggered by OM-motor 18 in RU 9, motor 6 in RU 3 can attach, generate force and spread thin filament activation through 11 monomers on either direction (line 2 in d). OM-motor 7 can attach and decrease the Z-ward spread of thin filament activation to 4 monomers (line 3 in d). Thus, binding of motor 7, being an OM-motor, prevents the propagation of the activation up to the actin monomer corresponding to motor 10, as it happens in control (lines 3-4 in b). The outcome of the example is that (*i*) the increased spatial frequency of motors in the ON state for the contribution of OM-motors anticipates the attachment/force generation by the first motor to pCa 7.7, inducing a leftward shift of the threshold of the force pCa-relation that explains the increase in pCa_{50}; (*ii*) at pCa 7 (panel d), the interposition of OM-motor reduces n_H by reducing the propagation of thin filament activation with respect to force-generating motors.

Solution								
(A)								
	Na_2ATP	$MgCl_2$	EGTA	HDTA	CaEGTA	Na_2CP	TES	Temp. (°C)
Rel_{12}	5.44	7.0	25	---	---	19.11	100	12
Act_{12}	5.46	6.76		---	25	19.49	100	12
Rel_{25}	5.44	7.0	25	---	---	19.11	60	25
Act_{25}	5.46	6.76		---	25	19.49	60	25
Rel_{35}	5.44	7.0	25	---	---	19.11	40	35
Act_{35}	5.46	6.76		---	25	19.49	40	35
Pre	5.45	6.93	0.1	24.9	---	19.49	100	5
(B)								
	Na_2ATP	$MgCl_2$	EGTA	Imidazole	KP	NaN_3	PMS	Glycerol
Skinning	2.5	2.5	5	10	170	---	0.1	---
Storage	2.5	2.5	5	10	170	5	---	50

Supplementary Table 1. Composition of the experimental solutions (A) and of the skinning and storage solutions (B). All concentrations are in mM except glycerol (v/v). ATP, adenosine 5'-triphosphate; EGTA ethylene glycol-bis (β - aminoethyl ether)-N,N,N',N'-tetraacetic acid; HDTA, 1,6 diaminohexane-N,N,N',N'-tetraacetic acid; TES, N-tris[hydroxymethyl]methyl-2-aminoethanesulphonic acid; CP, phosphocreatine disodium salt hydrate; KP, potassium propionate; PMSF, phenylmethylsulphonyl fluoride. 10 mM glutathione and 1 mg/ml creatine phosphokinase were added to the solutions in (A). 10 μM trans-epoxysuccinyl-L-leucylamido-(4. guanidino) butane (E-64) and 20 μg/ml leupeptin, were added to the solutions in (B). pH (adjusted with KOH) was 7.1 at the different temperatures. Rel_{temp} and Act_{temp}, relaxing (Rel) and activating (Act) solution at a given temperature (temp). Pre, preactivating solution at 5°C. Ionic strength ~190 mM; free Mg^{2+}, 1.7-1.9 mM and MgATP, 4.9-5.0 mM. All chemicals were obtained from Sigma (St. Louis, MO). 4% dextran (T-500, Alfa Aesar, Thermo Fisher Scientific) was added to the experimental solutions.

		T_0	F_0	n_H	pCa_{50}
soleus	df	3	3	3	3
	F-value	10.62	17.750	29.417	3.570
	P-value	$1.41*10^{-4}$	$3.40*10^{-6}$	$4.76*10^{-8}$	$2.96*10^{-2}$
Soleus 1 µM OM	df	2	2	2	2
	F-value	8.239	9.305	4.002	0.079
	P-value	$5.6*10^{-3}$	$3.63*10^{-3}$	$4.66*10^{-2}$	$9.24*10^{-1}$
psoas	df	2	2	2	2
	F-value	33.628	31.052	5.802	4.163
	P-value	$5.73*10^{-7}$	$1.03*10^{-6}$	$1.07*10^{-2}$	$3.17*10^{-2}$

Supplementary Table 2. Results of one-way ANOVA of the effects of temperature on the parameters reported in Table 1. df, degrees of freedom.

SI References

1. Gordon AM, Homsher E, Regnier M. Regulation of contraction in striated muscle. *Physiol Rev* **80**, 853-924 (2000).

2. Haselgrove JC. X-ray evidence for conformational changes in the myosin filaments of vertebrate striated muscle. *J Mol Biol* **92**, 113-143 (1975).

3. Huxley HE, Brown W. The low-angle x-ray diagram of vertebrate striated muscle and its behaviour during contraction and rigor. *J Mol Biol* **30**, 383-434 (1967).

4. Craig R, Offer G. Axial arrangement of crossbridges in thick filaments of vertebrate skeletal muscle. *J Mol Biol* **102**, 325-332 (1976).

5. Linari M*, et al.* Force generation by skeletal muscle is controlled by mechanosensing in myosin filaments. *Nature* **528**, 276-279 (2015).

6. Fusi L*, et al.* Minimum number of myosin motors accounting for shortening velocity under zero load in skeletal muscle. *J Physiol (London)* **595**, 1127-1142 (2017).

7. Debold EP, Romatowski J, Fitts RH. The depressive effect of Pi on the force-pCa relationship in skinned single muscle fibers is temperature dependent. *Am J Physiol Cell Physiol* **290**, C1041-1050 (2006).

8. Governali S*, et al.* Orthophosphate increases the efficiency of slow muscle-myosin isoform in the presence of omecamtiv mecarbil. *Nature communications* **11**, 3405 (2020).

9. Kampourakis T, Zhang X, Sun YB, Irving M. Omecamtiv mercabil and blebbistatin modulate cardiac contractility by perturbing the regulatory state of the myosin filament. *J Physiol (London)* **596**, 31-46 (2018).

10. Caremani M*, et al.* Modulation by Inotropic Interventions of the Regulatory State of the Cardiac Thick Filament in Diastole. *Biophys J* **118**, 593a (2020).
